# Benefits and Barriers to Emerging Technologies to Promote Health, Well-Being, and Independence of Older Adults

**DOI:** 10.1093/geroni/igab046.1022

**Published:** 2021-12-17

**Authors:** Walter Boot

## Abstract

Emerging technologies, such as voice assistant systems and artificial companion robots, hold a great deal of promise for improving the health, wellbeing, and independence of older adults. However, these solutions will likely be ineffective in the absence of research to understand barriers to the adoption and use of these technologies and without an exploration of the needs and preferences of older adults. This symposium focuses on both the potential of such technologies and factors that may affect their success. H. Spangler will present a detailed analysis of privacy concerns of older adults, with and without cognitive impairment, related to the use of Voice Assistant Systems (VAS). R. Nicholson will discuss the potential of a VAS app for promoting exercise among older adults and their caregivers to enhance mobility independence, with a focus on perceived benefits and dislikes about the app that may impact use. Finally, C. Berridge will present an exploration of perceptions of and attitudes toward artificial companion (AC) robots across the lifespan, before and after the start of the COVID-19 pandemic, including concerns about privacy. Together, these talks will highlight novel methods through which emerging technologies can support older adults and issues to consider if these methods are to produce meaningful change.

